# Muscle and Bone Health in Postmenopausal Women: Role of Protein and Vitamin D Supplementation Combined with Exercise Training

**DOI:** 10.3390/nu10081103

**Published:** 2018-08-16

**Authors:** Deborah Agostini, Sabrina Donati Zeppa, Francesco Lucertini, Giosuè Annibalini, Marco Gervasi, Carlo Ferri Marini, Giovanni Piccoli, Vilberto Stocchi, Elena Barbieri, Piero Sestili

**Affiliations:** 1 Department of Biomolecular Sciences, University of Urbino Carlo Bo, 61029 (PU) Urbino, Italy; deborah.agostini@uniurb.it (D.A.); sabrina.zeppa@uniurb.it (S.D.Z.); francesco.lucertini@uniurb.it (F.L.); giosue.annibalini@uniurb.it (G.A.); marco.gervasi@uniurb.it (M.G.); carlo.ferrimarini@uniurb.it (C.F.M.); giovanni.piccoli@uniurb.it (G.P.); vilberto.stocchi@uniurb.it (V.S.); piero.sestili@uniurb.it (P.S.); 2 Interuniversity Institute of Myology (IIM), University of Urbino Carlo Bo, 61029 (PU) Urbino, Italy

**Keywords:** postmenopausal women, sarcopenia, osteoporosis, exercise, dietary protein, vitamin D

## Abstract

Menopause is an age-dependent physiological condition associated with a natural decline in oestrogen levels, which causes a progressive decrease of muscle mass and strength and bone density. Sarcopenia and osteoporosis often coexist in elderly people, with a prevalence of the latter in elderly women. The profound interaction between muscle and bone induces a negative resonance between the two tissues affected by these disorders worsening the quality of life in the postmenopausal period. It has been estimated that at least 1 in 3 women over age 50 will experience osteoporotic fractures, often requiring hospitalisation and long-term care, causing a large financial burden to health insurance systems. Hormonal replacement therapy is effective in osteoporosis prevention, but concerns have been raised with regard to its safety. On the whole, the increase in life expectancy for postmenopausal women along with the need to improve their quality of life makes it necessary to develop specific and safe therapeutic strategies, alternative to hormonal replacement therapy, targeting both sarcopenia and osteoporosis progression. This review will examine the rationale and the effects of dietary protein, vitamin D and calcium supplementation combined with a specifically-designed exercise training prescription as a strategy to counteract these postmenopausal-associated disorders.

## 1. Introduction

Sarcopenia was firstly described at the end of the twentieth century by Rosenberg as a degenerative depletion in muscle mass [1] associated with age. It also involves the loss of muscle functionality leading to mobility restriction, functional impairment and physical disability [2] and finally loss of independence and reduced quality of life.

Even though there is still heterogeneity in diagnostic criteria and modalities to detect sarcopenia, the most used is the definition adopted by the European Working Group on Sarcopenia in Older People (EWGSOP) [3]. The group recommended, for the diagnosis of sarcopenia, that low muscle mass should be associated with low muscle function (defined as strength and performance) and proposed an algorithm for case finding in older individuals based on measurements of gait speed, grip strength and muscle mass. They also described measurement tools and specific age/gender cut off points to distinguish between presarcopenia, sarcopenia and severe sarcopenia [3]. Afterwards, an International Working Group on Sarcopenia (IWGS) incorporated sex-specific threshold values for muscle mass [4] while the Foundation of NIH (FNIH) Sarcopenia Project proposed a different definition for sarcopenia [5]. 

Although it is challenging to distinguish among sarcopenia, frailty and cachexia, they do represent different conditions: frailty has been defined by Morley et al. as “a medical syndrome with multiple causes and contributors that is characterized by diminished strength, endurance and reduced physiologic function that increases an individual’s vulnerability for developing increased dependency and/or death,” of which sarcopenia can be an aspect [6]; cachexia, characterized by weight and muscle mass loss, can be a cause of sarcopenia having a great inflammatory component [7].

The loss of muscle mass begins substantially at the age of 50 and continues afterwards [8] with similar gender-independent changes, such as increased inflammation and satellite cell senescence, reduced myocyte regeneration and protein synthesis [9] and several other gender-dependent alterations caused by the age-associated decrease of sex hormones [10]. Due to the decrease of testosterone in men and oestrogens in women, people of both genders experience sarcopenia. Although in general, men show a greater decay in muscle mass, women frequently present sarcopenia, since their muscle mass level in young age is physiologically much lower [11,12]. In a recent meta-analysis, Shafiee et al. examined the overall prevalence of sarcopenia in both women and men aged >60 years using the EWGSOP, IWGS and Asian Working Group for Sarcopenia criteria: they reported a prevalence of about 10% in adults, without global gender differences [13]. Hormonal replacement therapy (HRT) aimed at preventing the modifications and chronic somatic diseases caused by age-related oestrogen decrease, results in greater muscle strength in 50–65 years women, while in older women studies are not conclusive [14,15].

Several studies highlighted an association between sarcopenia and osteoporosis, another age-related disease involving low bone mineral density (BMD), bone tissue frailty and risk of fractures [16]. Osteoporosis is diagnosed by BMD criteria or occurrence of fragility factors [17]. Osteoporosis is more prevalent among older individuals with a far higher prevalence in women, where the onset often coincides with menopause. Indeed, it is estimated that the overall effect of menopause is an annual bone loss of about 2% during the first six years and 0.5–1% thereafter [18]. In western countries, the risk of osteoporotic fractures during the lifetime is about 40–50% in women and 13–22% in men [19]. Osteoporotic fractures often require hospitalisation and long-term care; thus, osteoporosis represents a significant health challenge worldwide. The Women’s Health Initiative is a long-term health study focused on strategies for preventing disease in postmenopausal women, aimed to analyse and suggest strategies to manage postmenopausal related problems effectively. During the project, eleven clinically risk factors have been identified, providing new insights into the epidemiology of osteoporosis [20].

Muscle-bone physiological interaction is increasingly reputed to be essential to prevent disease and disability in the elderly: in particular, Sjöblom et al. reported that women suffering from sarcopenia have more than a double higher risk of fracture and falls compared to those without the disease [21]. Among multiple factors, the musculoskeletal decline is also linked to protein, calcium and vitamin D availability and decrease in physical activity level. Deterioration in muscle and bone health is majorly caused by an inadequate protein intake associated with a significant demand due to an aging-related increase of protein anabolic resistance, chronic inflammation and oxidative processes [22]. The lack of physical activity, often affecting elderly people, accelerates muscle catabolism and is another major risk factor. Collectively, these problems may lead to a vicious cycle of muscle loss, injury and inefficient repair, causing elderly people to become progressively sedentary over time. Thus, therapeutic and/or nutritional strategies improving muscle mass and regeneration in the aged are nowadays required. Importantly, these strategies could also allow to maintain the capacity of sustaining and practicing physical exercise. Indeed, exercise is known to mitigate several deleterious effects of aging, such as insulin resistance, mitochondrial dysfunction and inflammation in muscle [23] and represent one of the best strategies to counteract sarcopenia. Resistance exercise is a trigger for muscle protein synthesis and can work in synergy with adequate protein intake [24].

In the elderly vitamin D deficiency often occurs and it is associated with sarcopenia, bone loss and disability. Vitamin D is highly interconnected with phosphate and calcium metabolism, as first demonstrated by Harrison and Harrison in 1961 [25]. The 1,25-dihydroxyvitamin D, D (1,25(OH)2D) the active metabolite of vitamin D, also known as calcitriol, increases intestinal phosphate absorption enhancing the expression of type 2b sodium–phosphate co-transporter [26,27]. Moreover, a deficiency of phosphate stimulates 1α-hydroxylase to convert vitamin D to calcitriol, which in turn stimulates phosphate absorption in the small intestine. Furthermore, calcitriol can also induce the secretion of Fibroblast-like growth factor-23 by osteocytes in bone, which lead to phosphate excretion in the kidney [28], as well as feedback on vitamin D metabolism. Since vitamin D is responsible for adequate intestinal absorption of calcium and phosphate, it maintains appropriate circulating concentrations of these minerals, which enable normal mineralization of the bone contributing to muscle health. Thus, adequate intake of calcium and vitamin D, associated with a correct lifestyle, is suggested during aging [29].

On the whole, the increase in life expectancy for postmenopausal women along with the need to improve their quality of life makes it necessary to develop specific therapeutic strategies, in association with HRT or as an alternative to it. Here we discuss the effect of protein intake, vitamin D supplementation, physical activity and of their synergistic administration in maintaining musculoskeletal health in postmenopausal women.

## 2. Mechanisms Involved in Muscle and Bone Loss in Postmenopausal Women

One of the most responsive pathways involved in musculoskeletal health is the Mammalian target of rapamycin (mTOR), involved in several anabolic processes in skeletal muscle [30]. mTOR is an evolutionarily conserved serine/threonine kinase known to play critical roles in protein synthesis. A better understanding of mTOR signalling in the maintenance of skeletal muscle mass might favour the development of mTOR-targeted treatments to prevent muscle wasting with particular attention at the healthy muscle in postmenopausal women conditions [31]. A well-known upstream stimulator of mTOR in skeletal muscle is insulin-like growth factor 1 (IGF-1), recognized as indispensable for muscle growth and regeneration [32,33,34] IGF-1 binds to the IGF-1 receptor (IGF1-R), a receptor tyrosine-kinase and subsequently recruits insulin receptor substrate-1. The specific role of each IGF-1 isoform and their post-translational modifications [35] must be taken into consideration for their effect in the proper tissue or microenvironment context. Furthermore, IGF-1 is directly involved in mitogenesis and neoplastic transformation, suggesting that this signalling pathway plays an important role in cancer promotion. IGF-1-therapeutic strategies must be viewed in the appropriate tissue context and in function of the IGF-1 circulating level and depending on IGFBP availability. 

In women, the age-related decline of skeletal muscle mass and strength accelerates with the beginning of menopause. Oestrogen signalling of muscle satellite cell activation and proliferation is mediated via oestrogen receptor-alpha (ERα) placed on skeletal muscle and activates several signalling pathways including IGF-1 signalling, nitric oxide signalling or activation of the phosphor-inositide-3 kinase/protein kinase B (Akt) pathway which then act to positively influence muscle satellite cells and promote protein synthesis [31]. Recent investigations demonstrated that IGF-1 and its receptor IGF1-R were not necessary for the induction of hypertrophy and the activation of Akt/mTOR in mechanical loading [36]. The expression of dominant negative (DN)-IGF-1 receptor specifically in skeletal muscle promoted muscle hypertrophy using an increased functional overload model induced by synergistic ablation [36]. Of notice, DN-IGF-I receptor-expressing muscle showed a comparable level of Akt and p70S6K1 activation. This data does not exclude an alternative upstream mediator for IGF1-R that could regulate Akt/mTOR signalling in skeletal muscle hypertrophy. In women, recent studies showed that the expression of IGF1-R in skeletal muscle cells increased in postmenopausal period after oestrogen replacement [37]. Moreover, it is known that oestrogen has an anabolic influence on muscle stimulating IGF-1R [38]. ERs are also expressed in human muscles [39]. In this regard, Wiik et al. have described the form of ERα and ERβ in both myonuclei and capillaries [40]. Their expression and distribution in muscle fibres appear greater in men, women and children, compared to postmenopausal women [40]. Notably, ERs can be also activated through IGF-1 that acts in stimulating their transcriptional activity [41]. Indeed, ERs could take part in muscle strength increase through the effect of both oestrogen and IGF-1. Despite that, both oestrogen and IGF-1 reduced at menopause, probably affecting muscle mass and strength.

Accordingly, estradiol plays an important role in the morphological muscle status increasing translocation of the glucose transporter, GLUT-4 to the plasma membrane through Akt pathway. Indeed, it causes an increase of myogenin and myosin heavy chain levels, which are important in skeletal muscles remodelling [42,43]. Estradiol also induces the Akt phosphorylation in myoblasts and its administration in postmenopausal women up-regulates the expression of mTOR genes [44]. As known, muscle wasting occurs when catabolic states overcome anabolic states. Sex hormones (i.e., androgens and oestrogen) play different roles in muscle mass maintenance and their decrease during aging negatively affects musculoskeletal health. Testosterone promotes an anabolic state activating protein synthesis and muscular regeneration through the androgen receptor, expressed in mesenchymal stem cells, satellite cells and fibroblasts [45]. Furthermore, it acts increasing circulating and intramuscular IGF-1 [46]. The catabolic state is promoted by the ubiquitin-proteasome system (UPS), autophagy-lysosomal system and apoptosis. Myostatin and inflammatory cytokines promote Forkhead box O (FOXO) protein activation that induces UPS and autophagy-lysosomal systems. Oestrogen is likely to promote an anti-inflammatory and anti-catabolic influence on muscle, especially after exercise, even though a complete characterization of mechanisms is lacking [10]. 

Several studies demonstrated an association between sarcopenia and osteoporosis, another age-related disease characterized by low BMD leading to bone tissue frailty and risk of fractures [16], with a higher prevalence in women. Biomechanical and biochemical interactions in the musculoskeletal unit are of great importance in the regulation and maintenance of tissue function. As functional units, muscles and long bones adapt to respond to metabolic and mechanical demand in health and they deteriorate together with ageing because of the same biomechanical and biochemical link between these two tissues [47]. The ‘mechanostat’ theory of Frost states that bone adjusts itself to sustain strain in a physiological window [48]: bone formation occurs if a greater strain is requested (i.e., physical activity), while lower strains (i.e., inactivity) will promote bone resorption. Accordingly, there will be an increase or a decrease, respectively, in muscle mass. Alongside biomechanical coupling in the musculoskeletal unit, also biochemical communication should be considered in muscle-bone crosstalk since both muscle and bone act as endocrine organs secreting respectively “myokines” and “osteokines” [49]. Skeletal muscle releases several hundred proteins and peptides capable of influencing bone health. The myokine [50] and osteokine [51] irisin, for example, is increased by exercise and has anabolic effects on muscle [50] and on osteoblast lineage by enhancing differentiation and activity of bone-forming cells [52]. Also, myostatin—that is, a negative regulator of muscle growth and interleukin-6 (IL-6)—is reported to have effects on bone [53]. Furthermore, skeletal muscle expresses high levels of several microRNAs that can be delivered by exosomes [54]. Information regarding the endocrine and paracrine effects of muscle-derived exosomes is limited but they are likely to play a role also in bone [53]. 

Also, tendons, ligaments, cartilage and connective tissue can affect muscle bone cross-talk [49]; periosteum, that separates muscle and bone, is semi-permeable and molecules such as IGF-1, IL-5 and prostaglandin E2 could permeate this membrane [55]. Taking into account the tight connection between muscle and bone, maintaining healthy skeletal muscles (i.e., through adequate exercise and nutrition) can help in counteracting osteoporosis in postmenopausal women.

Oestrogen-based HRT has an important role in maintaining and enhancing muscle mass and strength and also in protecting against muscle damage. The benefits of oestrogen for the skeletal muscle coupled with their additional positive actions on bone and metabolic health in older females provide further incentives for HRT use to enhance overall health in postmenopausal women. HRT is associated with an improved contractile function and power in 50–65-year-old women [15], while research is not conclusive in older, postmenopausal women. Analysing coronary heart disease and mortality, HRT showed many benefits in early observational data for use in younger healthy women (50–60 years) but age stratification revealed no benefit and increased harm in >60 year women, together with an increased breast cancer risk [56]. Recently the US Preventive Services Task Force recommended against the prevention of chronic condition in menopause using a combined oestrogen and progestin therapy and against oestrogen alone in postmenopausal women after hysterectomy [57], due to well documented harmful effects. Marjoribanks et al., in a systematic review on long-term HRT for perimenopausal and postmenopausal women, concluded that even though HRT is effective in osteoporosis prevention, it should be recommended as an option only when the risk of disease is very high and no other strategy is available [58]. They suggest that the adoption of HRT, if necessary, should be short-termed, provided that there is no increased risk of cardiovascular and thromboembolic disease and of several types of cancer [58]. The disadvantages related to the menopause, however, are not always manifested all at the same time and in all women; in some it seems not to be completely, in others there are only some disturbances, in others, finally, these disadvantages occur together and can also be very evident and frustrating. 

The risks associated with taking a pharmacological substitution therapy is much debated, but, in light of the most recent scientific acquisitions, menopause can be tackled by acquiring healthy habits that prevent the related disorders. Thanks to the better understanding of the causes, the ease of access to the diagnosis and the possibility of treatment before fractures occurrence today, a real prevention of both sarcopenia and osteoporosis and associated complications is possible. First of all, the fact is that muscle and bone health is a process that must develop throughout life in both males and females. Building a strong and healthy muscle-skeletal structure in childhood and adolescence can be the best defence. The further key steps that should be pursued at all ages for a successful prevention should consider: monitor a balanced diet rich in protein sources, calcium, magnesium and vitamin D that could interfere with anabolic mediators for both muscle and bone; practice exercise to enhance muscle strength, power output, neuromuscular activity and muscle mass; follow healthy lifestyles (avoiding alcohol, smoke and drugs) and, when appropriate, perform tests to define bone mineral density and possibly undergo appropriate treatment.

## 3. Exercise

Given the strict association between loss of muscle (namely, sarcopenia) and bone mass (namely, osteoporosis) that accompany aging, physical activity and exercise represent effective preventive and therapeutic strategies able to slow down sarcopenia progression and prevent/delay the onset of and treat, osteoporosis. Indeed, exercise has beneficial effects on muscle mass, muscle strength and physical performance [59,60,61], which counteract the reduced ability to perform activities of daily living and the increased risk of musculoskeletal injuries related to sarcopenia [62,63]. Exercise has also been shown to delay the onset of osteoporosis [64,65,66] and to improve balance [67] and muscular fitness [64,65,66,68] thus it is generally regarded as the primary non-pharmacological treatment for the prevention of osteoporosis and fall-related fractures. Since menopause occurs approximately with the onset of sarcopenia, aging non-physically active postmenopausal women should switch as soon as possible to an active lifestyle to prevent osteoporosis, while those already osteoporotic should exercise regularly to improve bone health and reduce the risk of fractures. It is well known that exercise, particularly progressive resistance exercise training (RET), is effective in increasing muscle mass, strength and endurance. Specific recommendations and guidance to prescribe exercise to treat sarcopenia, which update and extend those of the American College of Sports Medicine (ACSM) to promote muscle hypertrophy, strength and power [69], have recently appeared in literature [70]. Exercise that enhances muscle strength and mass also increases bone mass (i.e., bone mineral density and content) and bone strength of the specific bones stressed and may serve as a valuable measure to prevent, slow, or reverse the loss of bone mass in individuals with osteoporosis. Although further studies are still needed to determine optimal exercise prescription parameters for preventing osteoporosis and fractures [64,65,66] a recent consensus on physical activity and exercise recommendations for adults with osteoporosis [71] has stated the appropriateness of the current physical activity guidelines [68,72] for those without spine fractures and has proposed safer exercise guidance and strategies for those with a history of vertebral fractures. The ACSM’s framework for exercise prescription employs the so-called FITT-VP principle [73], which reflects the frequency (F), intensity (I), time (T) and type (T) of exercise and its volume (V) and progression (P) over time, in an individualized exercise training program. A detailed description of the FITT-VP principle for each type of exercise i.e., aerobic, resistance, flexibility and balance- adapted to postmenopausal ageing women according to the abovementioned studies, is provided in the following tables (Table 1, Table 2, Table 3 and Table 4).

Literature put a strong emphasis on resistance training for all individuals with osteoporosis [71] and recommend moderate to high intensity RET to treat sarcopenia [70]. Therefore, since preventing the loss of -or increasing- muscle strength and endurance is a cross-cutting goal for both sarcopenic and osteoporotic postmenopausal women, emphasis on progressive RET has been proposed. As a consequence of this approach, in this population there is the need to account for daily protein intake and, even, timing of protein supplementation [74].

## 4. Dietary Protein

Aged skeletal muscle possesses a reduced ability to respond to amino acid and insulin levels, leading to the concept of anabolic resistance, influenced by dietary protein digestion and amino acid absorption, plasma availability and hormonal response [75]. Moreau et al. reported differences in splanchnic protein metabolism during aging, with a maintained muscle protein synthesis (MPS), in a condition of an adequate rate of plasma levels of essential amino acids [76]. Alongside a reduced ability to use protein, a greater demand and a reduced intake are often present [22]. Chronic low-grade inflammation, that is often linked to oestrogen decrease and to visceral adipose tissue, favours proteolysis over synthesis and leads to an increased MPS demand [77]. Recommendations for dietary protein intake in general population with a moderate physical activity is 0.8 g of protein per kilogram of body weight per day [78]; however, due to increased protein demand in healthy older people, the European Society for Clinical Nutrition and Metabolism has proposed a daily recommended amount of 1.0–1.2 g/kg body weight/day as optimal for a healthy older individual [79,80]. Physical activity and exercise require a higher protein intake than sedentary condition [81].

Data from the National Health and Nutrition Examination Survey (NHANES) showed that, despite recommendations, daily protein intake decreases in the elderly and at least 8% of women consume an insufficient amount of protein [82]. 35% of institutionalized elderly people do not reach the recommended daily allowance (RDA) [83]. Gregorio et al. analysed the association of dietary protein amount with physical performance in postmenopausal women [84]. A sample of 387 healthy women has been studied, revealing an average consumption of 1.1 g/kg body weight/day and a percentage of 25% of subjects resulted below the RDA: they reported that subjects within the low protein group possess an impaired upper and lower extremity functionality than those in higher consumption group and that subjects with higher BMI and fat/lean ratio often consume protein below the RDA [84]. It should be remembered that higher fat mass is associated with impaired muscle metabolism [85] and insulin resistance, which is recognised as precursor of frailty [86].

Increased availability of amino acids has positive effects on muscle anabolism [87] improving lean body mass [88]. Protein intake also increases IGF-1 plasma concentration [89], together with muscle mass and strength [90]. In addition to the bone anabolic effect of IGF-1, increased protein intake has also been shown to reduce bone resorption [91]; furthermore, protein can act modifying calcitriol and intestinal calcium absorption, increasing bone health [92]. As explained, protein intake above RDA may be of benefits in postmenopausal women and Antonio et al. demonstrated the safety and effectiveness of three-fold higher dose in increasing lean mass, in both genders in association with resistance exercise [93]. In young women, the author also excluded a dangerous effect of high protein intake (more than 2.2 g/kg body weight/day for six months) on bone mineral content [94]. 

The best daily frequency for consuming protein, that is, single versus fractionated intakes, is still under debate. Indeed, some studies reported that daily protein should be consumed in a single meal, since a protein pulse feeding was more efficient than protein spread feeding in improving protein retention [95] and increasing plasma postprandial amino acid concentrations [96]. However, Kim et al. [97] found no differences comparing the effect of protein distribution pattern on functional outcome and protein kinetics; other studies reported that within-day protein distribution was more efficient in improving protein synthesis [98] and was negatively correlated with frailty [99]. Finally, a more frequent consumption of meals containing 30–45 g of protein [100] or protein supplementation at breakfast and lunch [101] have been recently associated with better lean mass preservation in older people.

Several factors can influence postprandial MPS: in particular, muscle disuse as a consequence of sarcopenia or immobilization causes a decrease in basal metabolic rate and muscle strength [102] and dietary protein consumption fails to act in this pathological condition [103]. On the contrary essential amino acid induces an enhanced effect on MPS if ingested following a resistance training session [104]. The mechanism involved in exercise-improvement of dietary protein effect depends on an increased amino acid delivery to the muscle through blood flow [105] and on mTOR pathway activation (after resistance exercise), that lead to muscle mass enhancement if an adequate amino acid pool is present: notably, this process seems to be delayed in elderly [106].

Alongside total protein amount and within-day protein distribution, quality of protein and their sources should be considered [107] in terms of essential amino acids and leucine content and of digestion/absorption kinetics. Pennings et al. [108] demonstrated that whey stimulates postprandial muscle protein accretion more effectively than casein and casein hydrolysate in older men, and attributed this effect to whey’s faster digestion and absorption kinetics and to higher leucine content. 

More recently, Zhu et al. demonstrated that in older postmenopausal well-nourished healthy women (70–80 years old) 30 g/day of extra protein did not improve the maintenance of muscle mass or physical function despite muscle deterioration in the upper limb. The authors attributed the lack of effects to the high habitual protein intake of women involved in this study, suggesting that protein intervention could be more effective in not well-nourished population. Furthermore, the intervention was not carried out in combination with resistance exercise, which has been demonstrated to improve protein effect [109]. Daly et al. reported that a protein-enriched diet equivalent to 1.3 g/kg body weight/day achieved through lean red meat consumption is safe and useful for enhancing the effects of resistance training on lean body mass and muscle strength and reducing circulating IL-6 concentrations in elderly women [110]. In this study, women were in a broader age range (60–90) and more than 40% had a history of HRT: these differences, in combination with training, could explain the discrepancies in muscle response.

Figueiredo Braggion et al. compared the effects of diets rich in vegetable protein versus animal protein in ovariectomized old female rats (a condition that may only mimic human menopause) in association or not with resistance training. They demonstrated that animal protein diet combined with training promoted muscle remodelling (reduction in type I and IIA fibres with an increase in type IIB fibres in medial gastrocnemius muscle, with increased collagen volume density) more efficiently compared to other conditions applied [111]. More importantly, a very recent human study showed that animal derived protein consumption, combined with physical activity, is positively associated with muscle mass and strength across ages in men and women [112].

Milk is a high-quality protein source, able of increasing muscle synthesis to a similar extent that whey [113] and beef [114]. However, the proposed protein amount of protein/meal of 30 g would require the consumption of one litre of milk [115]; for this reason, Orsatti et al. have recently proposed the addition of soy protein to milk to enhance the effect of resistance training in postmenopausal women muscle [116]. Soy represents a good alternative to animal products, even though it is less effective in promoting muscle protein synthesis than animal sources [117], due to the high amount of isoflavones. Isoflavones are, in turn, often used as a natural alternative to hormone therapies and have been demonstrated to reduce the loss of bone mass and inflammation [118] that occur in menopause. Orsatti et al. observed that the addition of soy protein to milk, in association to resistance exercise, improves muscle strength but not muscle mass and attributed the latter to leucine content, that is lower with respect to the values suggested for maximizing protein synthesis [116].

Besides the effect of global amino acid availability, specific amino acids such as leucine, glutamine and arginine can play an important muscle health effect. Leucine (an essential amino acid) supplementation has been proposed as a strategy to counteract anabolic resistance in older muscle since it acts as a signalling molecule able to activate mTOR and thus protein synthesis [119]. Also, Xia et al. suggested an increase of leucine consumption in the diet, together with concurrent training, to counteract sarcopenia associated with chronic low-grade inflammation, often present during menopause [77]. Glutamine and arginine can also differentially regulate mTOR [120,121]. The nonessential amino acid glycine has anti-inflammatory and antioxidant properties and seems to promote the preservation of muscle mass. In a mice model of inflammation, glycine has been demonstrated to counteract anabolic resistance, since the improvement in leucine-stimulated protein synthesis was accompanied by higher phosphorylation status of mTOR, ribosomal protein S6 kinase and eukaryotic translation initiation factor 4E binding protein 1 compared with L-alanine-treated controls [122].

Taken together, the above evidence suggests that postmenopausal women need an adequate protein intake, in association with exercise (according to the modalities described in Table 1, Table 2, Table 3 and Table 4) to counteract sarcopenia and related bone loss.

## 5. Vitamin D 

Vitamin D is known to significantly contribute to the regulation of calcium and phosphorus homeostasis and skeletal mineralization through endocrine effects on bone, intestine, parathyroid glands and kidney [123]. Vitamin D has both skeletal and extra-skeletal beneficial effects. There is growing evidence that vitamin D regulates many other cell functions and its potential effect on skeletal muscle mass and strength is receiving greater attention. The biological actions of vitamin D on muscle cell differentiation, metabolism and function may be multiple, acting through direct and indirect, genomic and non-genomic pathways.

Vitamin D can be cutaneous synthesized from 7-dehydrocholesterol (7-DHC) (80–90%), a precursor of cholesterol, after exposure to ultraviolet B light. This endogenous synthesis mainly depends on the intensity of solar radiation. Very limited number of foods contains vitamin D such as fatty fish (like salmon and mackerel) or mushrooms, whereas milk products and eggs contain only small amounts of vitamin D. Calcitriol can be produced both by dietary sources and endogenous vitamin D through two hydroxylation reactions. The diet provides about 10–20% of the daily requirement of vitamin D. 25-hydroxyvitamin D (25(OH)D), the precursor of calcitriol, is the major circulating form of vitamin D and is considered the best biomarker to assess the vitamin D status; it circulates bound to a specific plasma carrier protein, vitamin D binding protein, that also transports the calcitriol.

The Institute of Medicine defines plasma concentration of 25(OH)D as adequate (25(OH)D concentrations >  50 nmol/L or > 20 ng/mL), insufficient (25(OH)D concentration between 30–50 nmol/L or 12–20 ng/mL), or as deficient (25(OH)D levels <  30 nmol/L or < 12 ng/mL). The committee stated that 50 nmol/L is the serum 25(OH)D level that covers the need of 97.5% of the population. Serum concentrations >125 nmol/L or > 50 ng/mL are associated with potential adverse effects [124].

Serum 25(OH)D levels < 50 nmol/L are associated with increased bone turnover, bone loss and possibly mineralization defects and poorer outcomes for frailty, hip fracture and all-cause mortality [29]; furthermore, it may exacerbate osteoporosis in elderly or postmenopausal women by increasing the rate of bone turnover. Aging decreases the capacity of human skin to produce vitamin D, in particular, 7-DHC concentration, the precursor of vitamin D, declines in the elderly [125]. During aging, the combined effect of a decline in intestinal calcium absorption, in the ability of the kidney to synthesize calcitriol and an increase in its catabolism contributes to age-related bone loss [123]. In addition, with aging there is a defect in 1 α hydroxylation [123].

High levels of dietary calcium intake and/or calcium supplements can significantly improve bone mineral content and density in postmenopausal women, however, some studies suggest that calcium supplements alone may not be sufficient to reduce fracture risk and that additional vitamin D supplementation is required; calcium, in combination with vitamin D supplementation, reduces the risk of fragility fractures and increases the survival in the elderly [126,127].

Furthermore, an increased level of calcium intake during the period of childhood and adolescence can lead to a reduction in the risk of osteoporosis during old age and post menopause [128].

Vitamin D has a pivotal role in the regulation and uptake of calcium in muscle cells, promoting protein synthesis and calcium and phosphate transport in muscle, which is important for muscle strength and contractile activity. Vitamin D appears to optimize the effect of dietary protein on skeletal muscle anabolism [29]. Both direct and indirect effects of vitamin D seem to play a role in muscle functionality, although most of them are attributed to the concomitant hypocalcaemia and hypophosphatemia [128]. Vitamin D plays a key role in regulating calcium-dependent functions of muscle, such as contraction, mitochondrial function and insulin sensitivity [128]. Loss of muscle mass is related to vitamin D deficiency [129,130]. The mechanisms by which vitamin D affects muscle strength and function have not yet fully clarified but are likely mediated by the vitamin D receptor (VDR); VDR and 1-alpha hydroxylase are both expressed in muscle tissue [131].

The presence of nuclear VDRs in muscle tissue suggests that vitamin D acts on muscle via a genomic transcriptional effect. Mechanistically, it has been suggested that 1,25-dihydroxyvitamin D binds to the nuclear VDR in muscle resulting in de novo protein synthesis [132]. Salles et al. have reported an anabolic effect of vitamin D in murine C2C12 myotubes through an increased insulin receptor and VDR mRNA expression [133]. Both the transcriptional induction of these genes and the enhancement of the insulin and leucine action on the related protein is one of the cardinal processes of vitamin D effect on skeletal muscle anabolism [133].

Furthermore, vitamin D signalling via VDR regulates gene transcription and activates further intracellular signalling pathways involved in calcium metabolism and it has been suggested to be involved in myoblast proliferation and differentiation [134]. Proximal myopathy (proximal weakness), characterizes patients with VDR-dependent rickets, an evidence arisen from studies in either older or younger populations [128]. Additionally, VDR-knockout mice are characterized by abnormal muscle morphology/physical function, while VDR polymorphisms have been associated with differences in muscle strength [135]. Muscle and bone VDR and 1-alpha hydroxylase expression decrease with aging [131,136,137] and it might be involved in intramuscular inflammation, since it has been associated with an increase of IL-6 and TNF-alpha levels in human skeletal muscle [138]. This process leads to the inhibition of muscle protein synthesis, to skeletal muscle apoptosis [139] and increased differentiation of myogenic precursor cells into adipocytes [140]. Recently, several authors have demonstrated that vitamin D supplementation can modulate VDR expression [131]. From a pathogenic point of view, reversible atrophy of type II muscle fibres and fatty infiltration of skeletal muscles have been reported in patients with vitamin D deficiency [131,141]. In younger adults, serum 25(OH)D concentration is inversely related to muscle fat infiltration, independently from body mass index and physical activity [142]. Such changes in muscle lipid content may have important implications for musculoskeletal function. Hence, since low vitamin D status is common in many elderly populations [143], attention should be paid to the potential therapeutic benefits of its supplementation. To this regard, European guidance for the diagnosis and management of osteoporosis in postmenopausal women recommends a daily intake of at least 1000 mg/day for calcium, 800 IU/day for vitamin D and 1 g/kg body weight of protein for all women aged over 50 years [29]. On the whole, vitamin D deficiency is associated with a loss of muscle mass and strength in elderly people and with a decline in physical performance. A nutritional intervention of vitamin D and amino acid supplementation could be a strategy to support muscle protein availability and synthesis in sarcopenia condition.

## 6. Conclusions

**Figure 1:**
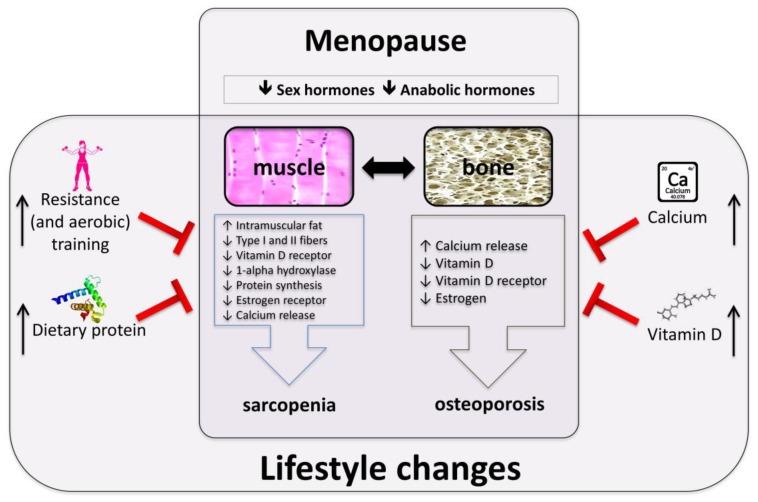
Menopause-related factors affecting muscle and bone and their possible prevention through a rationale strategy based on protein and vitamin D supplementation regimens in combination with specifically-designed training protocols.

Osteoporosis and sarcopenia are two disorders affecting elderly people; their increasing incidence, due to the longer life expectancy in most western countries, might become an uncontrolled clinical and financial burden in the next few years. Early diagnosis, prevention and treatment of these disorders represent a very current but actually unmet, social and medical need. The evidence of profound interactions between bone and muscle causes a sort of negative resonance between the two tissues when they are simultaneously affected by osteoporosis and sarcopenia, respectively. Indeed, the coexistence of this twin condition in ageing leads to an accelerated worsening of the quality of life, poor clinical perspectives and high utilization of health resources. Due to the age- and/or gender-associated prevalence of sarcopenia and osteoporosis, postmenopausal women are potentially more prone to such a joint clinical situation. Nutritional and lifestyle factors may positively affect muscle and bone mass and function and have the advantage to be cheap and safe. To this regard, protein, vitamin D and calcium supplementation combined with a specifically-designed training protocol, emphasizing progressive RET, are capable of directly targeting some of the major physio-pathological causes of the twin condition progression and could simultaneously and coherently delay or revert the vicious cycle leading to the reciprocally-induced deterioration and wasting of osteoporotic bone and sarcopenic muscle (Figure 1).

The medical and social relevance of strategies alternative to HRT targeting both sarcopenia and osteoporosis progression based on a female-specific rationale would be invaluable. To this regard, the development of controlled and selected protein and vitamin D supplementation regimens in combination with specifically-designed exercise training protocols may represent a cheaper and safer alternative to oestrogen replacement therapies.

## Figures and Tables

**Table 1:** Aerobic (cardiorespiratory endurance) exercise recommendations for ageing postmenopausal women.

Intensity—I	Frequency—F	Time—T (Duration)	Type—T (Mode) [Examples]	Volume—V (Quantity)	Progression—P (Rate of)	Specific Notes
**Moderate:** 40–59% of VO_2_R or HRR; 64–75% HR_max_; 4–5 RPE	At least 5 day∙week^−1^	30 to 60 min each session (i.e., at least 150 min∙week^−1^)	Weight-bearing activity [walking, jogging, dancing, or other activities where full body weight issupported by limbs]	≥500–1000 MET∙min∙week^−1^	Increase gradually any of the FITT components (as tolerated). Initiate increasing exercise duration: an example is adding 5–10 min every 1–2 week over the first 4–6 week and adjusting upward over the next 4–8 months to meet the recommended FITT components	If tolerated, moderate to vigorous intensity and 3–5 day∙week^−1^ frequency is recommended but lower intensities and frequencies are still beneficial when the current physical activity level is low. For individuals with a history of vertebral fracture vigorous intensity may not be appropriate because it might increase the risk of falls or fractures: in those patients, moderate intensity is recommended
**Vigorous:** 60–89% of VO_2_R or HRR; 76–95% HR_max_; 6–8 RPE	At least 3 day∙week^−1^	20 to 60 min each session (i.e., at least 75 min∙week^−1^)				

Modified from [69,70,72]. MET∙min: metabolic equivalents (MET) of energy expenditure for a physical activity performed for a given number of minutes (min), calculated as MET × min; VO_2_R: oxygen uptake reserve, calculated as the difference between maximal oxygen uptake and resting oxygen uptake; HRR: heart rate reserve, calculated as the difference between maximal heart rate and resting heart rate; HR_max_: maximal heart rate; RPE: rate of perceived exertion, on the 0–10 scale.

**Table 2:** Resistance (strength) exercise recommendations for ageing postmenopausal women.

Intensity—I	Frequency—F	Time—T (Duration)	Type—T (Mode) [Examples]	Volume—V (Quantity)	Progression—P (Rate of)	Specific Notes
**Novice exercisers:** ∼8 to 12 repetitions performed near task failure (i.e., ∼10 to 14-RM or 5–8 on the 0–10 RPE scale)	1–2 day∙week^−1^	Depends on exercise volume (number of sets, repetitions for each set and rest intervals in-between) and is not associated with effectiveness	Any form of movement designed to improve muscular fitness by exercising a muscle or a muscle group against external resistance: exercise and breathing techniques are of paramount importance [free weights, resistance machines, weight-bearing functional tasks, etc.]	1 set of 8–12 repetitions (no more than 8–10 exercises per session)	Progress with small increments possible [e.g., 2–10% 1-RM, depending on muscular size and involvement, is recommended]. If a break is taken, lower the level of resistance by 2 weeks’ worth for every week of no exercise	Avoid making absolute restrictions about amount of weight allowed, instead place emphasis on safe movement recommendations; avoid rapid, repetitive, weighted, or end-range flexion or rotation of the spine; avoid lifting from or lowering to the floor; avoid exercises to improve strength/endurance in “core” or “abdominal” muscles involving repeated flexion or rotation of the spine (isometric exercises, or holds are preferable). In individuals with a history of vertebral fracture a consultation with an exercise specialist/therapist with training in exercise prescription for osteoporosis is highly recommended (in the absence of such consultation, it may be advisable to limit resistance exercises to those that use body weight, the floor, or the wall to provide resistance)
**Intermediate to experienced exercisers:** ∼8 to 12 repetitions performed to task failure (i.e., ∼8 to 12-RM or >8 on the 0–10 RPE scale)	2–3 day∙week^−1^			2 sets of 8–12 repetitions (no more than 8–10 exercises per session)		

Modified from [69,70,72]. RPE: rate of perceived exertion, on the 0–10 scale; 1-RM: one repetition maximum, that is, the load that can be lifted one time only; multiple RM: the load that can be lifted no more than the specified times.

**Table 3:** Flexibility (stretching) exercise recommendations for ageing postmenopausal women.

Intensity—I	Frequency—F	Time—T (Duration)	Type—T (Mode) [Examples]	Volume—V (Quantity)	Progression—P (Rate of)	Specific Notes
Stretch to the point of feeling tightness or slight discomfort	≥2–3 day∙week^−1^ (stretching on a daily basis is most effective)	Hold a static stretch for at least 10–30 s (30–60 s may confer greater benefit). Accumulate a total of 60 s of stretching for each flexibility exercise by adjusting time/duration and repetitions (see volume) according to individual needs	Stretching exercise that increase the ability to move a joint through its complete ROM (provided individual specific conditions are accounted for) (static active flexibility; static passive flexibility; dynamic flexibility; ballistic flexibility; proprioceptive neuromuscular facilitation; etc.)	Repeat each exercise 2–4 times in order to attain the goal of 60 s stretch time [e.g.: two 30-s stretches or four 15-s stretches]. A stretching routine can be completed approximately in ≤10 min	Optimal progression is still unknown	Focus on joints with low ROM. Flexibility exercises are most effective when the muscles are warm

Modified from [69,70,72]. ROM: range of motion.

**Table 4:** Balance exercise recommendations for ageing postmenopausal women.

Intensity—I	Frequency—F	Time—T (Duration)	Type—T (Mode) [Examples]	Volume—V (Quantity)	Progression—P (Rate of)	Specific Notes
Not applicable	Daily	≥15–20 min	Exercises include those that reduce the base of support in static stance [e.g., semi-tandem, tandem, or one-legged stand], a dynamic or three-dimensional balance challenge [e.g., Tai Chi, tandem walk, walking on heels or toes], or other strategies to challenge balance systems [e.g., weight shifting, reduced contact with support objects, dual-tasking, close eyes during static balance challenges, etc.]	Cumulative time: 2 h *per* week	Progress from “standing still” to “dynamic” exercises. Progression of the balance challenge should occur over time [e.g., moving to a more difficult exercise, removing vision or contact with support object, or dual-tasking, etc.]	Balance can be exercised during daily walks or activities, while standing still reduce the base of support, semi-tandem stance, one-leg stand; shift weight between heels and toes or during dynamic movements [e.g., Tai Chi; tandem walk, dancing, etc.]

Modified from [69,70,72].
